# Transcranial Doppler ultrasound validation of BOLD‐fMRI cerebral blood flow relationship

**DOI:** 10.1002/mrm.70091

**Published:** 2025-09-17

**Authors:** Genevieve Hayes, Joana Pinto, Sierra Sparks, Daniel P. Bulte

**Affiliations:** ^1^ IBME, Department of Engineering Science University of Oxford Oxford UK

**Keywords:** BOLD fMRI, cerebral blood flow, cerebrovascular reactivity, hypercapnia, transcranial Doppler ultrasound

## Abstract

**Purpose:**

A precise understanding of the interplay between cerebral blood flow (CBF) and blood oxygen level‐dependent (BOLD) fMRI signals is essential for advancing cerebrovascular research. Although calibrated BOLD approaches often rely on arterial spin labelling (ASL) to estimate CBF, alternative validation using transcranial Doppler ultrasound (TCD) has not been explored. This study aims to determine whether a simplified hemodynamic model and linear regression can accurately characterize the relationship between TCD‐derived CBF velocity and BOLD‐fMRI responses during a ramp CO_2_ stimulus. We hypothesized that both models would provide robust fits within the moderate partial pressure of end‐tidal carbon dioxide (PETCO_2_) and BOLD signal ranges tested.

**Methods:**

Twenty‐five healthy participants underwent two sessions. In session 1, right middle cerebral artery velocity (MCAv) was acquired using clinical TCD. In session 2, 3 T BOLD‐fMRI data were collected. Both sessions used a ramp PETCO_2_ protocol with deep breaths followed by 5% and 10% CO_2_. Data processing included motion correction, spatial smoothing, fieldmap correction, high‐pass filtering, and PETCO_2_ alignment with smoothed MCAv (MCA $\bar{v}$) and BOLD signals from the right parietal lobe. A simplified hemodynamic model and linear regression were applied to assess the MCA $\bar{v}$‐BOLD relationship, with model performance evaluated by R^2^.

**Results:**

Final analysis included 21 participants. The hemodynamic model produced consistent fits (R^2^ ≥ 0.69). Linear regression showed strong agreement between MCA $\bar{v}$ and BOLD (R^2^ = 0.759).

**Conclusion:**

Both modeling approaches successfully linked TCD‐derived MCA $\bar{v}$ and BOLD‐fMRI responses during hypercapnia. These findings support the use of TCD as a complementary surrogate for CBF in BOLD calibration and cerebrovascular research.

## 1 INTRODUCTION

Understanding the relationship between cerebral blood flow (CBF) and BOLD fMRI signal is critical for cerebrovascular research. Because pioneering work demonstrated the BOLD contrast mechanism, BOLD‐fMRI has become a widely used tool for mapping brain function in both clinical and research settings.^1^, ^2^, ^3^, ^4^ However, although increases in metabolic activity (e.g., neuronal oxygen consumption) often accompany neural activation, interpreting changes in the BOLD signal solely in terms of these metabolic processes is difficult. This is because the BOLD signal itself arises from a complex interplay among CBF, cerebral blood volume (CBV), and the cerebral metabolic rate of oxygen consumption (CMRO_2_).^5^, ^6^

These parameters jointly determine the ratio of hemoglobin in the imaging voxel, a critical factor because hemoglobin can exist as paramagnetic deoxyhemoglobin or diamagnetic oxyhemoglobin.^7^ Consequently, the concentration of deoxyhemoglobin influences the magnetic susceptibility within and around blood vessels, modulating the MRI signal.^4^ At rest, approximately 30% to 40% of the oxygen is extracted across the capillary bed, creating a substantial level of deoxyhemoglobin in venous and capillary vessels.^8^, ^9^ During neural activation, CBF increases more than CMRO_2_, leading to a decrease in deoxyhemoglobin concentration and an increase in the measured BOLD signal.^5^, ^6^ Meanwhile, CBV changes and volume exchange effects further complicate the relationship.

To facilitate the quantitative interpretation of this BOLD signal, the Davis model (also known as the deoxyhemoglobin dilution model) was introduced, where calibrated fMRI methods can be used to estimate underlying metabolic and hemodynamic parameters.^10^, ^11^, ^12^ The classical Davis model provides a relationship between changes in CMRO_2_, CBF, and the BOLD signal in fMRI,^10^, ^11^ as shown in Eq. ([1):

(1) $$\frac{\Delta \text{BOLD}}{\mathrm{BOL}D_{0}}=M\left[1-{\left(\frac{\mathrm{CBF}}{\mathrm{CB}F_{0}}\right)}^{\alpha -\beta }{\left(\frac{\mathrm{CMR}O_{2}}{\mathrm{CMR}O_{2|0}}\right)}^{\beta }\right],$$

where *M* is the scaling factor representing the maximum possible BOLD signal change under ideal conditions (dependent on field strength, baseline oxygenation, and vascular architecture, typically 8% to 12% in gray matter), α describes the relationship between CBF and venous CBV (typically 0.2–0.4), β describes the coupling between CBF and CMRO_2_ in terms of oxygen extraction fraction (typically 1.0–1.5 at 3 T).^10^, ^13^, ^14^, ^15^, ^16^, ^17^

During a ramp hypercapnia protocol, CBF is changed while maintaining CMRO_2_ relatively constant, and therefore, the last term of Eq. (1) will be very close to 1. This has been approximated in Eq. (2):

(2) $$\frac{\Delta \text{BOLD}}{\mathrm{BOL}D_{0}}=M\left[1-{\left(\frac{\mathrm{CBF}}{\mathrm{CB}F_{0}}\right)}^{\gamma }\right],$$

where γ=α−β describes the relationship between CBF, CBV, and CMRO_2_, essentially characterizing the contribution of CBF to the BOLD signal. This assumes an isometabolic response to hypercapnia, although it should be noted that this is still an area of controversy,^18^ with literature indicating reduced,^15^, ^19^, ^20^ unchanged,^14^, ^21^ and increased^22^ CMRO_2_ with hypercapnia.

Typically, calibrated BOLD experiments use arterial spin labeling (ASL) to measure CBF.^5^, ^23^, ^24^ ASL is a non‐invasive technique that allows quantification of regional CBF by magnetically labeling arterial blood water as an endogenous tracer. Accurate measurement of CBF via ASL requires accounting for factors such as tagging efficiency, blood T_1_ changes, and transit delays.^5^, ^23^ During ramp hypercapnia protocols, these parameters may vary dynamically with each change in the partial pressure of end‐tidal carbon dioxide (PETCO_2_), especially arterial transit time (ATT), which is sensitive to both CO_2_‐induced vasodilation and blood flow changes. Multi‐delay ASL can partially address this but increases acquisition time and complexity.^25^ As a result, ASL sequences during ramped or rapidly changing stimuli require careful optimization and may face reduced temporal resolution relative to transcranial Doppler (TCD) or BOLD.

Despite the wide adoption of ASL‐based approaches, independent validation of the Davis model using alternative methods for measuring CBF is still missing. Previous studies have compared BOLD and ASL‐derived cerebrovascular reactivity under hypercapnia, showing that BOLD and CBF measures are generally correlated, but can diverge depending on vascular tone and the nature of the stimulus.^13^, ^25^, ^26^ Several alternative approaches to calibrated BOLD modeling have also been proposed, including models incorporating direct venous oxygenation measurements to improve CMRO_2_ quantification.^27^ TCD ultrasound, which measures blood flow velocity in the major cerebral arteries, might provide a promising non‐invasive technique to complement and cross‐check BOLD‐fMRI findings. TCD is less sensitive to ATT variability and provides continuous velocity measurements during ramp protocols, although it assumes relatively stable vessel diameter for interpreting velocity changes as proportional to flow. TCD has been extensively used to assess cerebrovascular reactivity, but to date, there has been no direct validation of the Davis model—or simplified variants thereof, assuming constant CMRO_2_—against TCD‐derived flow velocity measures.

Here, we aim to address this gap by presenting the first validation of the simplified Davis model using TCD measurements of flow velocity in the middle cerebral artery (MCA), offering insights into the robustness of BOLD‐fMRI quantification of CBF changes. The primary objective of this study is to evaluate whether the Davis model can accurately characterize the relationship between TCD‐derived MCA blood velocity and BOLD‐fMRI signals during a controlled ramp CO_2_ stimulus. We hypothesize that the TCD blood velocity and BOLD responses will exhibit a consistent relationship that can be captured by the Davis model, under the assumption of constant CMRO_2_, because of possible plateauing of the BOLD signal at high PETCO_2_ and CBF levels. In addition, to evaluate whether this relationship can also be described by a simpler formulation, we fit a separate linear regression model to the same data to test the linearity of the BOLD‐TCD relationship within a moderate PETCO_2_ and BOLD signal range. Prior studies^14^, ^15^, ^16^ indicate that a linear model can also provide a good fit in this regime, where BOLD signal changes remain within an approximately linear portion of the CBF‐BOLD response curve.

To test these hypotheses, we acquired TCD and BOLD‐fMRI data during identical ramp hypercapnia protocols in healthy adults and performed model fitting and intermodal comparisons. By combining these two approaches, we seek to deepen our understanding of the physiological underpinnings of the BOLD signal and advance methods for cerebrovascular quantification in fMRI research.

## 2 METHODS

### 2.1 Data acquisition

**FIGURE 1:**
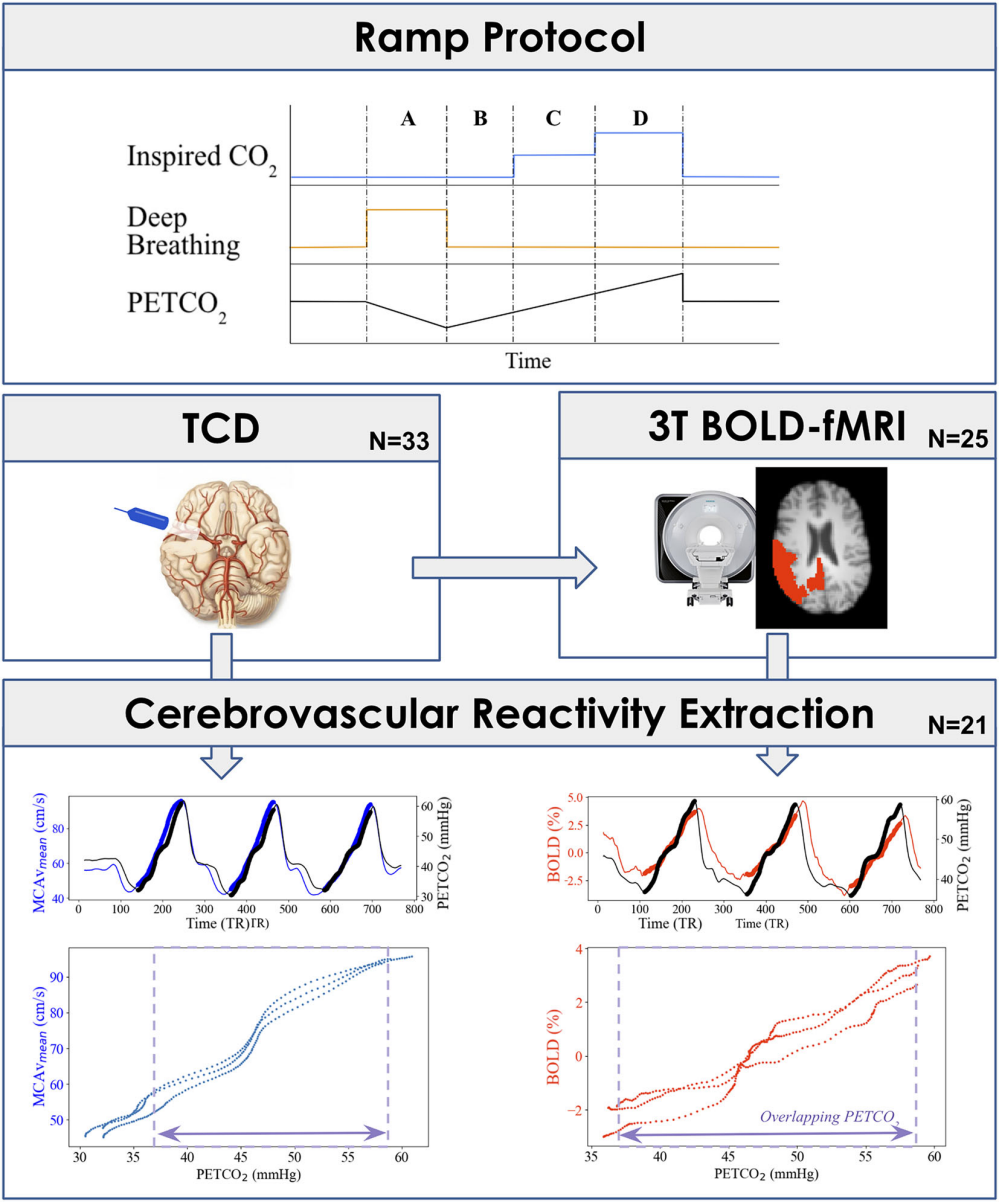
Diagram of the data collection and preprocessing steps for the transcranial Doppler ultrasound (TCD) and blood oxygen level‐dependent functional MRI (BOLD‐fMRI) in response to the ramp partial pressure of end tidal carbon dioxide (PETCO_2_) protocol. The ramp protocol consisted of three repetitions of (A) five deep breaths, followed by (B) 30 s of regular breathing on synthetic medical air, (C) 40 s breathing a 5% CO_2_ balanced gas mixture, and (D) 40 s breathing a 10% CO_2_ balanced gas mixture. Analysis of the smoothed TCD middle cerebral artery velocity (MCAv_mean_) and normalized percent BOLD change are presented for a representative subject.

All procedures conformed to institutional research ethics standards and with the Declaration of Helsinki. Twenty‐five healthy participants (13 females, 33 ± 11 years) underwent two separate sessions (19 ± 18 days apart). An overview of the acquisition and preprocessing pipeline is presented in Figure 1. In the first session, TCD ultrasound (7760EN Doppler‐BoxX Digital, Compumedics DWL) was used to measure blood velocity in the MCA using a 2 MHz probe. With the participant lying supine, the TCD probe was placed on the transtemporal window with transmission gel and was secured using an adjustable headset. The location and angle of the probe was changed until a consistent blood velocity profile was achieved. In the second session, BOLD‐fMRI data were acquired with a 3 T Siemens Prisma scanner (gradient echo [GE] EPI sequence, TR/TE = 800/30 ms, multi‐band = 6, volumes = 769, multislice interleaved, FOV = 216 × 216 mm^2^, resolution = 2.4 mm isotropic, 60 slices, flip angle = 52°) along with a high‐resolution MPRAGE anatomical image (TR/TE = 1900 ms/3.97 ms, FOV = 192 × 192 mm^2^, resolution = 1 mm isotropic).

A ramp PETCO_2_ protocol^28^ was used in both sessions during which inspired gases were delivered using a custom gas delivery system built in‐house at the University of Oxford where respiratory gas mixtures could be delivered one at a time at 15 L/min. The ramp protocol consisted of three repetitions of five deep breaths, followed by 30 s of regular breathing on synthetic medical air (21% O_2_/79% N_2_), 40 s breathing a 5% CO_2_ balanced gas mixture (BOC Group, Linde), and 40 s breathing a 10% CO_2_ balanced gas mixture (BOC Group, Linde).^28^, ^29^ Participants had 60 s of recovery time breathing medical air between each ramp. The gases were controlled manually during the TCD acquisition and the participants were notified verbally when to take their deep breaths and when the gases were changed. For the MRI acquisition, an automated valve controller for the gas delivery was used along with a visual cue to notify participants to take their deep breaths, both triggered by the MRI scanner. End‐tidal gases were collected through a face mask for accurate PETCO_2_ measurement. Additional details of the data acquisition and preprocessing steps are provided in Hayes et al.^30^

### 2.2 Data preprocessing

Data processing and analysis was performed using FSL 6.0^31^ and custom Python scripts. Smoothed TCD MCA blood velocity signals (MCA $\bar{v}$) were calculated using a rolling mean filter with a 5‐second window (result assigned to the right edge of the window index) to reduce the pulsatile cardiac signal, followed by low‐pass filtered to remove high‐frequency noise components above 2 Hz. MCA $\bar{v}$ was then normalized by dividing the smoothed blood velocity by the baseline blood velocity for each subject. The BOLD‐fMRI data underwent standard corrections in FSL, including motion correction (MCFLIRT tool), fieldmap correction (FUGUE tool), spatial smoothing (FWHM = 4 mm), and high‐pass temporal filtering (275 s).^32^, ^33^ Using FSL's FEAT toolbox, general linear modeling was used to select active voxels with a Z‐score >3.1 and a corrected cluster significance threshold of *p* = 0.05 using Gaussian random field theory as implemented by FSL's cluster tool.^34^ The mean BOLD signal was extracted from the right parietal region, using the MNI152 brain atlas, to align with the vascular territory of the MCA assessed by TCD.^35^, ^36^ The BOLD signal was then shifted so that zero BOLD signal corresponded to the baseline period. This ensured that BOLD responses were expressed as the BOLD signal change relative to baseline (as opposed to relative to the average BOLD signal) since the period with elevated PETCO_2_ (hypercapnia) was longer than the period with PETCO_2_ below baseline breathing (hypocapnia). This definition is consistent with the TCD signal processing. Finally, to account for respiratory delays in each modality, the corresponding PETCO_2_ trace was time‐shifted relative to the TCD or BOLD signal using cross‐correlation. For a detailed account of these steps, see Hayes et al.^30^

### 2.3 Data analysis

The aligned TCD MCA $\bar{v}$ versus BOLD‐fMRI data were fit with the simplified Davis model (Eq. [[2]) with fixed values of γ, solving only for the optimal M parameter using least squares regression. Values of γ = −1.2, −1.1, −1.0, and −0.9 were used based on values found in previous literature.^10^, ^14^, ^15^, ^16^, ^17^ In addition, linear regression was performed between MCA $\bar{v}$ and BOLD signals to test whether the relationship between modalities followed an approximately linear trend within the moderate PETCO_2_ and BOLD signal range used here. Prior work suggests that the BOLD‐CBF relationship is approximately linear within lower to mid‐ranges of BOLD signal change (˜6% to 8%) before saturating at higher levels.^14^, ^15^, ^16^ Therefore, a linear model served as a benchmark against which to compare the more complex Davis model fits and to assess whether BOLD signal changes tracked proportional changes in MCA $\bar{v}$ across our stimulus range.

### 2.4 Statistics

Paired 2‐tailed t tests were performed to compare baseline PETCO_2_ and peak PETCO_2_ values between the TCD and MRI sessions. These tests assessed whether there were significant differences in PETCO_2_ measurements across modalities, and the resulting t‐statistics and *p*‐values are reported. The t‐statistic quantifies the difference between the means of two related groups, normalized by the variability of their differences, and is used to determine whether the observed difference is likely because of chance. We considered results statistically significant at *p* < 0.05.

Goodness of fit of the simplified Davis model and linear model were assessed using the coefficient of determination, R^2^, where R^2^ < 0.4 indicates a poor fit, 0.4 ≤ R^2^ < 0.6 indicates a moderate fit, 0.6 ≤ R^2^ < 0.8 indicates a good fit, and R^2^ ≥ 0.8 indicates a very strong fit.^37^, ^38^

## 3 RESULTS

Data from four participants were excluded, two because of significant noise, and the two others because of high signal variability between sessions. Although only one participant was excluded in our prior modality‐specific study using this data,^30^ the three additional datasets were excluded here because of excessive variability and poor alignment between the TCD and BOLD responses within the overlapping PETCO_2_ range, which compromised intermodal model fitting. The mean BOLD signal was extracted from the right parietal region to align with the vascular territory supplied by the right MCA assessed by TCD. This region of interest (ROI) was functionally defined by applying a general linear model with PETCO_2_ as the regressor, and voxels within the MNI152‐defined right parietal lobe that showed significant activation (Z > 3.1, cluster‐corrected *p* < 0.05) were included. On average, 15 140 ± 1169 voxels were included per participant. Across all subjects included in the analysis (*n* = 21), the mean change in PETCO_2_ from the bottom of the ramp (minimum) to peak hypercapnia (maximum) was 27.8 ± 4.0 mm Hg in TCD and 27.6 ± 4.9 mm Hg in MRI (t‐statistic = 0.175, *p* = 0.863). The mean baseline PETCO_2_ during the TCD and MRI was 39.7 ± 3.7 mm Hg and 43.3 ± 3.7 mm Hg, respectively (t‐statistic = −6.299, *p* < < 0.01). To align the MRI and TCD signals, the overlapping PETCO_2_ range was selected which, on average, ranged from 35.9 ± 4.1 mm Hg to 59.6 ± 4.9 mm Hg. Within this overlapping PETCO_2_ range, the average MCA $\bar{v}$ across subjects ranged from 81% ± 7% to 175% ± 19% of baseline and the average BOLD signal ranged from −1.4% ± 1.1% to 4.9% ± 1.0%, with a maximum of 6.5% BOLD.

**FIGURE 2:**
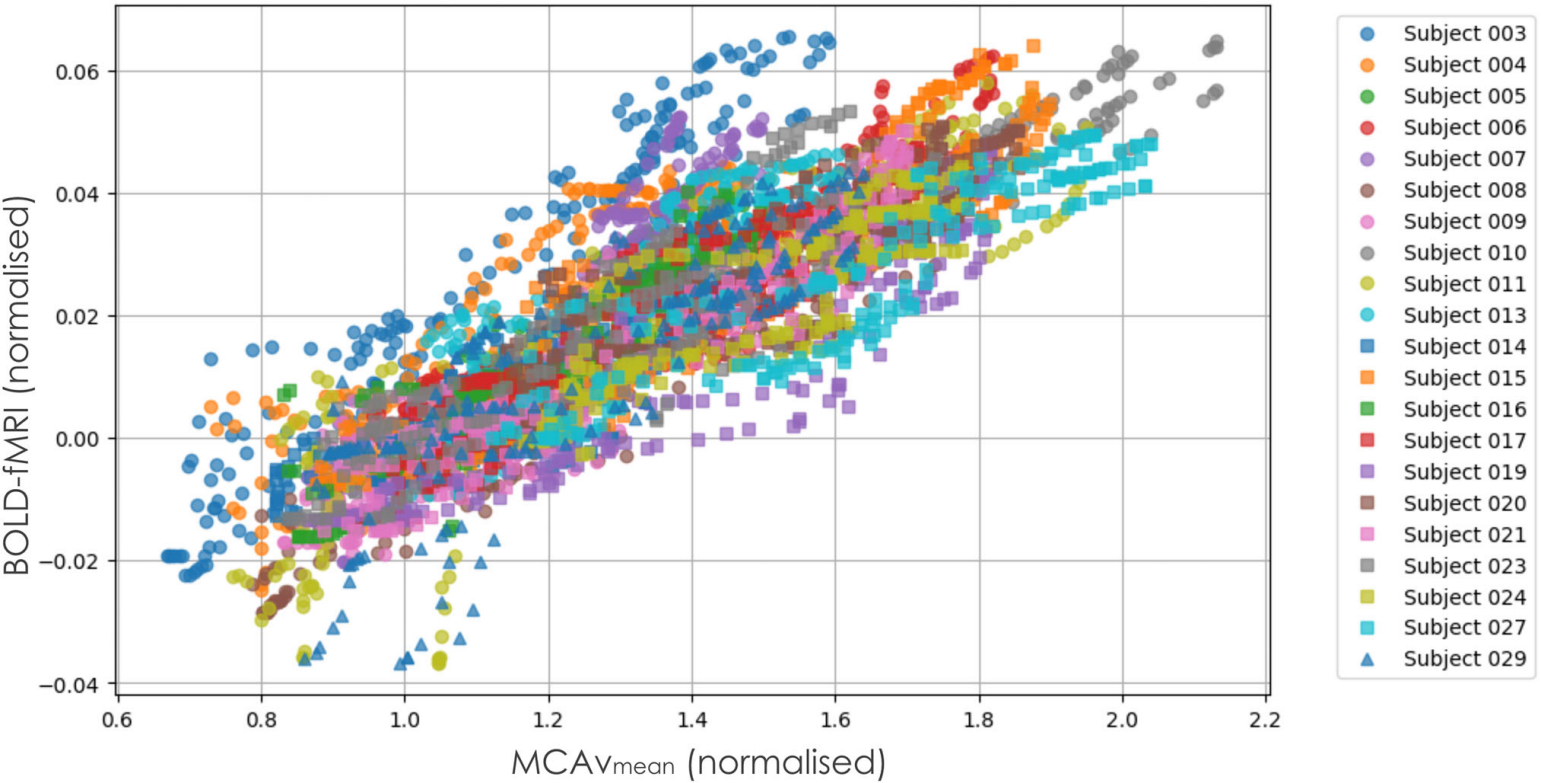
Scatterplot of normalized MCAv_mean_ (mean blood velocity in the middle cerebral artery) versus normalized BOLD signal change in the right parietal lobe for each subject (*n* = 21). Each dot represents a single partial pressure of end‐tidal carbon dioxide (PETCO_2_) point from the ramp CO_2_ challenge, with subjects shown in different colors and markers.

The combined BOLD‐MCA $\bar{v}$ data from all included participants is presented in Figure 2, with each subject's data shown in a unique color. The general trend is consistent across participants, although noticeable variability emerges at higher MCA $\bar{v}$ and BOLD values, reflecting inter‐subject differences in cerebrovascular reactivity. Because of this variability, particularly in the upper range of PETCO_2_ and flow, model fitting was performed using pooled data across participants. This enabled robust evaluation of whether the BOLD signal approached saturation at higher flow levels. Individual curve fitting was not feasible because of insufficient data density and greater noise within single‐subject responses. Table 1 reports individual minimal and peak PETCO_2_ values within the overlapping range from both modalities, along with maximum TCD and BOLD responses per participant.

**TABLE 1:** Participant‐specific values for overlapping PETCO_2_ ranges, and the maximum normalized MCA $\bar{v}$ (TCD) and maximum BOLD signal change (%) corresponding to the peak PETCO_2_.

Participant ID	Overlapping minimum PETCO_2_ (mm Hg)	Overlapping peak PETCO_2_ (mm Hg)	Max MCA $\bar{v}$ (normalized)	Max BOLD (%)
003	33	63	1.59	6.54
004	36	62	1.54	4.44
005	35	53	1.48	3.80
006	33	58	1.82	6.24
007	39	66	1.50	5.24
008	38	63	1.80	4.38
009	39	53	1.44	3.18
010	36	61	2.13	6.47
011	30	61	1.95	5.80
013	43	63	1.60	4.63
014	31	45	1.59	2.80
015	30	59	1.90	6.40
016	40	65	1.50	4.03
017	34	60	1.72	4.75
019	33	56	1.82	4.80
020	43	63	1.85	5.08
021	35	59	1.70	5.01
023	32	57	1.74	5.34
024	40	59	1.78	4.44
027	42	61	2.04	4.94
029	33	64	1.64	4.32
Mean ± SD	36 ± 4	60 ± 5	1.72 ± 0.19	4.89 ± 1.00

*Notes*: “Overlapping” refers to the PETCO_2_ range common to both modalities, used in all model fitting and signal comparisons. Data are presented per participant (IDs anonymized) along with group mean ± SD in the bottom row.

Abbreviations: PETCO_2_, partial pressure of end‐tidal carbon dioxide; MCA $\bar{v}$, middle cerebral artery velocity; TCD, transcranial Doppler; BOLD, blood oxygen level‐dependent signal.

**FIGURE 3:**
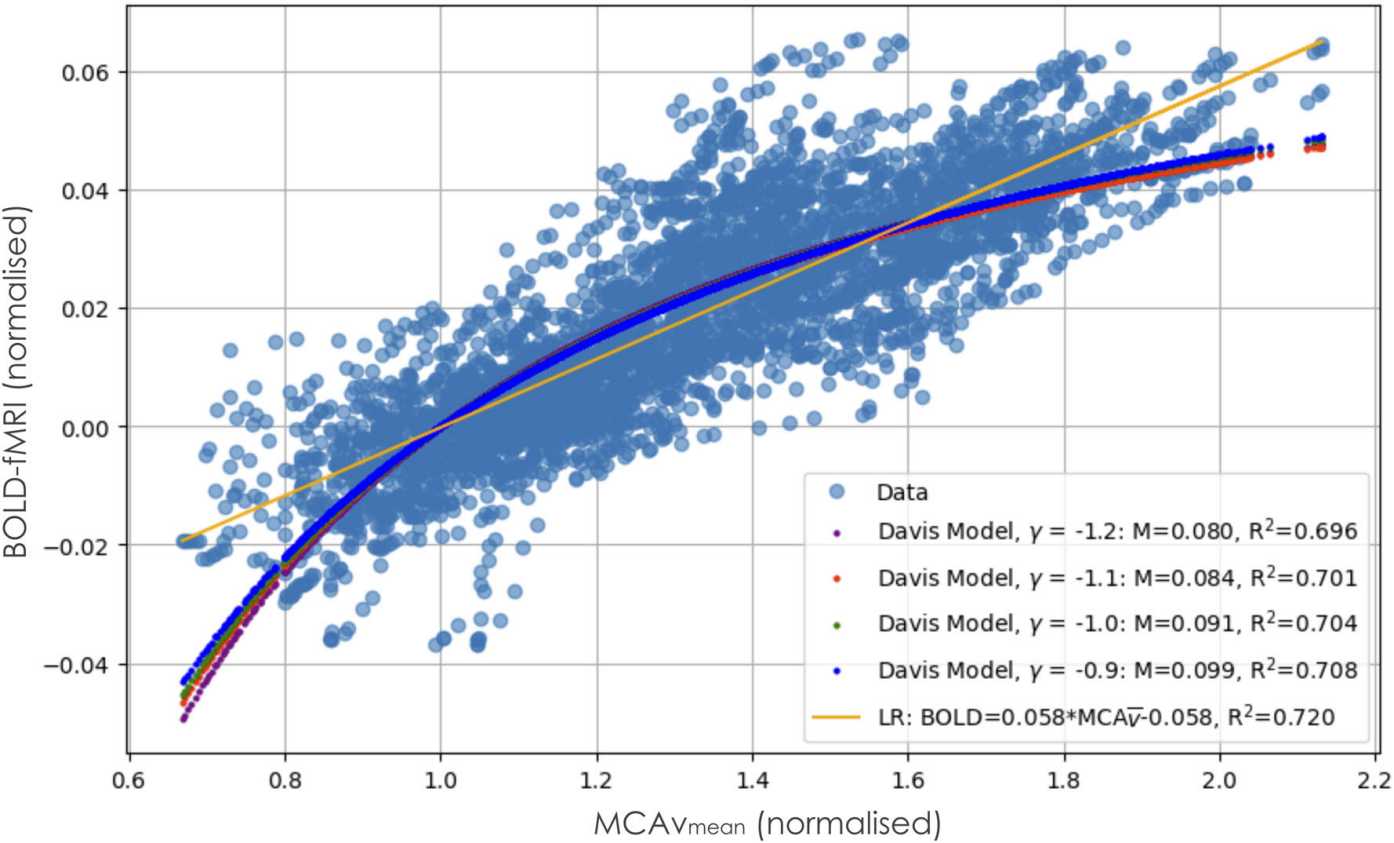
Group‐level best fits of the Davis model with fixed γ parameters and corresponding M parameters optimized by least‐squares regression. A linear regression (LR) of normalized BOLD signal as a function of normalized middle cerebral artery velocity (MCA $\bar{v}$) is also presented. The coefficient of determination (R^2^) for each fit is presented in the legend.

**FIGURE 4:**
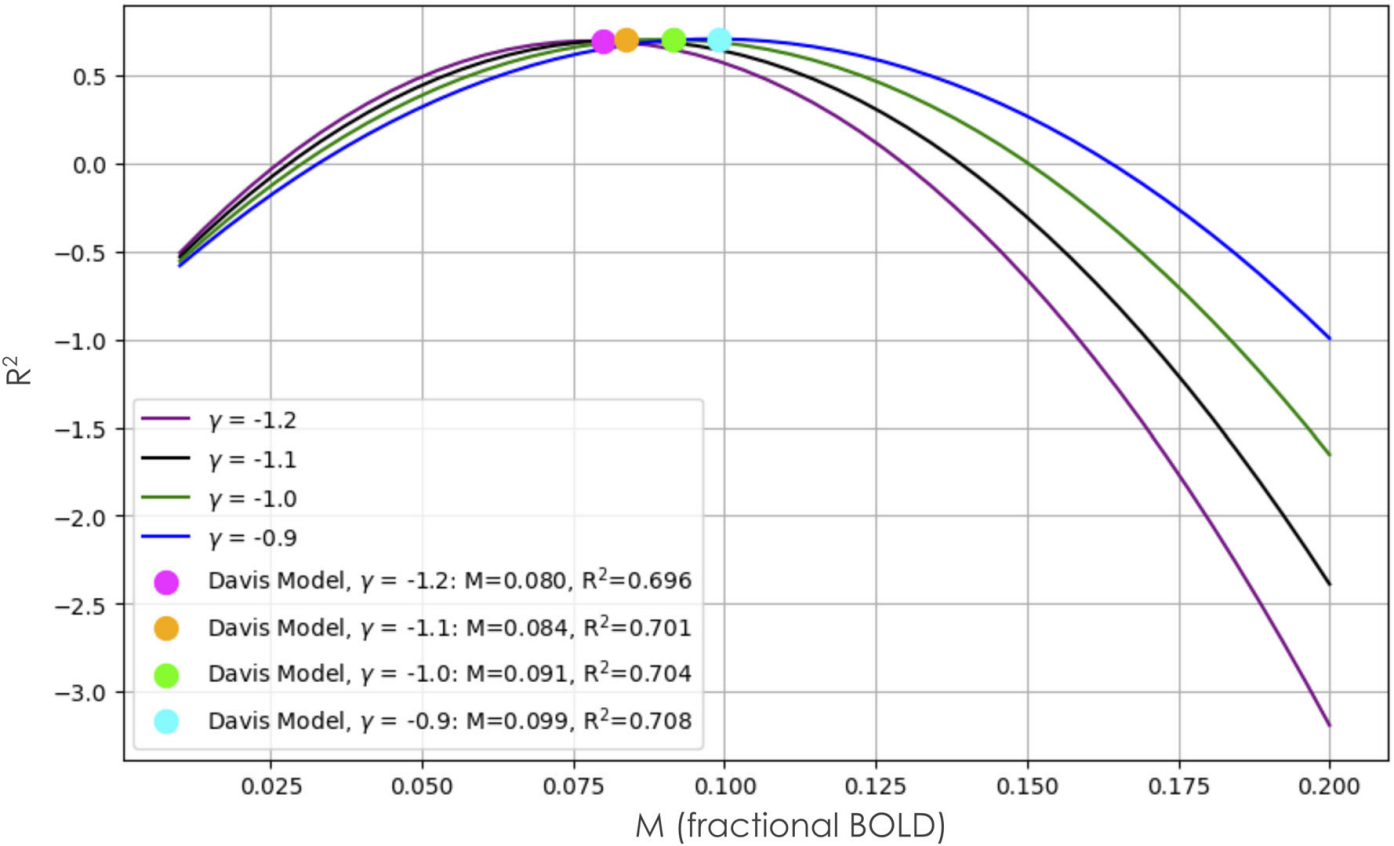
Coefficient of determination (R^2^) as a function of the M parameter at fixed γ of −1.2, −1.1, −1.0, and − 0.9. The best fit M parameters are highlighted with their corresponding parameters in the legend.

The best fit curves of the linear regression and the Davis model with γ fixed at −1.2, −1.1, −1.0, and − 0.9 on top of the combined subject data are presented in Figure 3. The optimal M for each γ is 0.080, 0.084, 0.091, and 0.099, respectively. The R^2^ as a function of M for each fixed γ is presented in Figure 4. The R^2^ of the best fits are 0.696, 0.701, 0.704, and 0.708, respectively, all corresponding to good fits to the data. The linear regression resulted in an R^2^ of 0.759. All models were constrained to pass through the baseline point (1.0, 0.0).

## 4 DISCUSSION

The principal finding of this study is that the simplified Davis model^10^, ^11^ provides a robust framework for characterizing the relationship between BOLD‐fMRI responses and changes in CBF, as proxied by TCD measures of blood velocity in the MCA. By investigating four different γ values (−1.2, −1.1, −1.0, and −0.9), drawn from previous literature,^10^, ^14^, ^15^, ^16^, ^17^ we obtained consistent and “good” fits (R^2^ = 0.696, 0.701, 0.708, and 0.708, respectively).

Interestingly, a simple linear regression between MCA $\bar{v}$ and the BOLD signal produced an R^2^ of 0.720—slightly higher than the Davis model fits. We expected that much of the BOLD‐MCA $\bar{v}$ data would follow a linear relationship based on prior literature showing that the BOLD‐CBF response is approximately linear within moderate stimulus ranges.^14^, ^15^, ^16^ However, it was somewhat surprising that this linear trend extended into the upper portion of our observed BOLD range (˜6.5%), still outperforming the Davis model fits. This may be because we remained below the BOLD saturation region, typically expected at 8% to 12% BOLD signal change at 3 T.^10^, ^13^, ^14^, ^15^, ^16^ Therefore, in this physiological regime, linear regression appears well‐suited to capture the dominant behavior of the BOLD‐MCA $\bar{v}$ relationship.

Figure 2 helps visualize the range of individual responses to the ramp CO_2_ protocol. Although a strong group‐level relationship is apparent, some participants showed flatter slopes or more scattered data, especially near the extremes of the response. The overlaid linear and Davis model fits in Figure 3 show similar performance at the group level, reinforcing the conclusion that both models capture the dominant relationship between MCA $\bar{v}$ and BOLD in this physiological range. The decision to combine data across participants for model fitting reflects both the consistent average trend and the variable sampling and noise at the individual level. Future studies with longer ramps or denser sampling could enable more robust subject‐level curve fitting and assessment of inter‐individual differences in cerebrovascular reactivity (CVR).

Our results align well with prior work on BOLD calibration and the Davis model. In the original formulation, Davis et al. suggested a method to quantify the interplay between the BOLD signal, CBF, and the CMRO_2_. Subsequent studies refined the model parameters, particularly the parameters that go into the γ exponent, highlighting its sensitivity to different field strengths and stimuli.^11^, ^13^, ^14^, ^15^, ^16^, ^39^, ^40^ The γ values tested here span the range reported in these refined calibrations, and our findings support the validity of using these literature‐derived values even when the assumption of constant CMRO_2_ is used.

The choice of γ is an important consideration in BOLD calibration. Based on our results, all γ values tested (−1.2 to 0.9) yielded similarly robust model fits and consistent M estimates across participants. This reflects prior literature suggesting that γ varies primarily with field strength, sequence parameters, and vascular compartment contributions.^10^, ^11^, ^13^, ^14^, ^15^, ^16^, ^17^, ^39^, ^40^ For future studies using 3 T BOLD‐fMRI with hypercapnia, we would recommend testing within this γ range unless more specific sequence‐calibrated estimates are available. In ramp hypercapnia protocols where BOLD signal changes remain below saturation (˜8%–12%), our results suggest that small deviations in γ have minimal impact on model performance.

A small but statistically significant difference in baseline PETCO_2_ was observed between the MRI and TCD sessions. This offset is likely because of a combination of the environment and experimental conditions. The MRI scanner environment, including the head coil and restricted space within the MRI bore, may have limited natural chest expansion or altered breathing depth compared to the more open setup used during TCD measurements. Additionally, the enclosed and low‐stimulation conditions of the MRI suite may promote increased relaxation or mild drowsiness, reducing respiratory rate and leading to elevated PETCO_2_ levels. Differences in room ventilation and gas circuit lengths may also have contributed. Despite this baseline discrepancy, the overall PETCO_2_ change across the ramp protocol did not differ significantly between modalities. Furthermore, all modeling was performed using data aligned to the session‐specific PETCO_2_ traces and constrained to the overlapping PETCO_2_ range. Therefore, this baseline difference is unlikely to bias the estimated relationships between PETCO_2_, MCA $\bar{v}$, and BOLD responses. Normalization to baseline values within each session also helps mitigate any confounding influence of absolute PETCO_2_ offsets on the results.

Our approach underscores the utility of TCD measurements as a rapid means of estimating flow changes in large intracranial arteries during hypercapnia challenges. Although TCD‐based velocity signals cannot directly capture local microvascular changes, prior research has demonstrated that MCA $\bar{v}$ can correlate with more direct measures of CBF across a range of physiological conditions. Studies using phase‐contrast MRI,^41^ resting ASL MRI,^42^ quantitative magnetic resonance angiography with a hypercapnic challenge,^43^ and single photon emission computed tomography (SPECT) in patients with cerebral vasospasm^44^ have shown moderate to strong correspondence between TCD‐derived MCA $\bar{v}$ and volumetric flow. However, this relationship is not universally observed. For example, Pearson et al.^45^ found significant correlation between positron emission tomography (PET)‐derived CBF and MCA $\bar{v}$ only half of the time in patients with internal carotid artery occlusion, likely reflecting regional flow redistribution and impaired autoregulation in disease states. Similarly, Burley et al.^46^ reported no consistent correlation between BOLD‐fMRI and TCD responses during fixed 5% CO_2_ inspiration in both elderly and young adults, possibly because of inter‐individual variability in ventilatory responses and cerebrovascular reactivity. These findings highlight that although TCD offers a valuable tool for assessing flow changes, its interpretability as a surrogate for CBF may depend on participant health status, experimental conditions, and regional vascular dynamics. The present work contributes to this body of evidence by linking TCD responses to BOLD signal changes within the Davis model framework and using linear regression.

Although this study used a ramp hypercapnia protocol, the Davis model and linear relationships identified here are theoretically applicable to block‐type CO_2_ paradigms, assuming similar physiological responses. However, differences in stimulus timing and sampling density may influence model fitting, and dedicated validation in block or step designs is warranted. Prior work has successfully used block hypercapnia designs to calibrate BOLD responses with ASL‐based CBF measurements.^14^, ^15^, ^47^ However, compared to ramp protocols, block designs provide fewer discrete PETCO_2_ points and may make it more challenging to constrain the full BOLD‐CBF response curve, particularly near saturation or at low PETCO_2_ levels. Additionally, ramp protocols offer smoother transitions and more data points across a continuous range of PETCO_2_, facilitating better model fitting and potentially more sensitive detection of subtle nonlinearities. Therefore, although we expect the general findings to hold across different stimulus designs, the precision of model parameter estimates may depend on the choice of protocol.

### 4.1 IMPACT OF THE RESEARCH

To our knowledge, this study is the first to test the simplified Davis model using TCD‐derived flow velocity measurements. By showing that the fitted M parameter (ranging from 0.080 to 0.099) remains stable across the tested γ values, we provide further support for the robustness and reliability of the Davis model in capturing hypercapnia‐induced changes in BOLD signal. Our work indicates that for ramp CO_2_ stimuli with an average change in PETCO_2_ of 28 mm Hg, both the simplified Davis model and a linear model can accurately describe BOLD responses, which will be of interest to researchers who require a less resource‐intensive methodology than full ASL‐based calibrated fMRI.

Moreover, these findings are directly relevant to clinical and translational cerebrovascular research. TCD is widely available in many clinical settings and provides a relatively inexpensive, bedside tool for assessing hemodynamic changes. Demonstrating that TCD can be coupled with BOLD‐fMRI, even in separate sessions, to yield consistent physiological inferences, expands the potential for multi‐modal validation of fMRI‐based CBF measurements.

### 4.2 LIMITATIONS AND FUTURE DIRECTIONS

Despite these promising results, several limitations must be acknowledged. TCD only quantifies blood flow velocity in a single large artery (the MCA in our case) rather than whole‐brain or region‐specific.^48^ Additionally, TCD assumes relatively stable vessel diameter, which may not perfectly hold at extreme levels of vasodilation. TCD and BOLD‐fMRI data were also collected on different days, 19 ± 18 days apart, with one participant having up to 83 days between sessions to accommodate participant and scanner availability, potentially introducing intra‐subject variability (e.g., as a result of sleep, stress levels, hydration status).^49^, ^50^ Although simultaneous measures of TCD and BOLD‐fMRI are not currently feasible because of technical limitations, future research could mitigate intra‐subject variability by reducing the time between sessions. Furthermore, future studies may benefit from replacing the assumption of isometabolic response to hypercapnia with additional direct measures.^19^, ^23^

The integration of ASL into this analysis would offer more direct quantification of CBF, however, its application during ramp hypercapnia is complicated by dynamic changes in ATT and other confounds, requiring multi‐delay or high‐temporal‐resolution sequences,^25^ which can increase scan time and complexity. Comparisons between BOLD and ASL‐derived CVR^13^, ^26^ suggest that these modalities can yield complementary information, but are not fully interchangeable. Future studies incorporating ASL alongside TCD and BOLD during ramp protocols would provide valuable additional insight and cross‐validation of methods.

Another promising technique is phase‐contrast (PC) MRI, which provides spatially resolved velocity measurements in large arteries and, when combined with cross‐sectional area estimates, can yield volumetric flow data. PC‐MRI has been used to assess single‐vessel cerebral blood velocity and flow dynamics.^51^, ^52^ However, PC‐MRI generally provides lower temporal resolution than TCD and is more susceptible to motion artifacts during dynamic stimuli, making it less ideal for capturing the full time course of ramp hypercapnia responses. Importantly, like TCD, PC‐MRI focuses on single‐artery flow and does not provide regional brain‐wide maps of CVR as BOLD‐fMRI does. Future work combining PC‐MRI, TCD, and BOLD during ramp protocols would further clarify the relationships between flow velocity, volumetric flow, and BOLD responses.

Although the ramp CO_2_ protocol elicited a range of responses that facilitated robust model fitting, variability between subjects in the CVR curve complicated the estimation of parameters such as M and γ without prior physiological constraints. As shown in Figure 2, individual trajectories differ in slope, curvature, and signal quality, particularly in the upper PETCO_2_ range. Variability in the CVR curve shape and amplitude could reflect differences in vascular anatomy, baseline flow/metabolism, or sensitivity to CO_2_, introducing biases to M and γ on an individual basis, particularly in the absence of independent physiological constraints such as ASL‐derived CBF or direct CMRO_2_ measures. For this reason, we elected to pool data across participants to capture group‐level physiological trends and assess for potential signal saturation. Although this enabled reliable model fitting, it limits the interpretability of subject‐specific CVR metrics. Future studies incorporating these complementary data and/or increasing the length or resolution of the ramp protocol to acquire more data could refine the calibration of model parameters across subjects. Notably, to define an “optimal” γ, future work could combine direct CBF (e.g., ASL or PC‐MRI) and CMRO_2_ measures with BOLD data in the same individuals, enabling a more rigorous calibration of the Davis model parameters under varying physiological conditions. Additionally, spatial variability in M across the brain—because of differences in vascular density, baseline oxygen extraction fraction, cerebral blood volume, and magnetic susceptibility—is an important consideration for interpreting regional CVR or calibrated BOLD analyses. In this study, our TCD‐based approach provides whole‐vessel velocity approximations and does not explicitly address regional M variability. Notably, we estimated M only in the gray matter of the right parietal lobe, aligned with the MCA vascular territory, where variability is expected to be lower in the gray matter of healthy adults under isometabolic conditions.^40^ Inclusion of spatial M maps would be informative for future studies seeking to assess regional heterogeneity.

Although the underlying physiological models may be generalizable, it remains an open question how well these findings, particularly the model parameter estimates and intermodal agreement, may translate to alternative hypercapnia paradigms such as block or step designs, which differ in temporal resolution and data sampling density. Although the Davis model has been validated in block designs using ASL,^14^, ^15^, ^47^ the continuous nature of the ramp protocol used here provided dense sampling of the BOLD and MCA $\bar{v}$ response across a wide PETCO_2_ range. Block paradigms, although compatible with ASL‐based CBF quantification, may offer fewer data points and could make fitting the full BOLD‐CBF response curve more challenging. Future comparative studies using matched block and ramp protocols would help elucidate how stimulus design impacts model parameter estimates and intermodal agreement.

A larger cohort would improve statistical power and the generalizability of the estimated parameters. The number of participants included in the analysis (*n* = 21) was modest and future research with larger cohorts is warranted to confirm these findings and to further explore physiological factors contributing to inter‐individual variability in cerebrovascular responses.

## 5 CONCLUSIONS

Our findings demonstrate that the simplified Davis model, anchored by literature‐based γ values, offers a strong fit to both BOLD and TCD velocity signals under a ramped hypercapnia protocol. The findings demonstrate reliable modeling of BOLD signal changes up to 6.5% BOLD signal change, with the strong linear fit supporting BOLD‐fMRI as a robust CBF estimate. This “proof‐of‐concept” technical validation underscores the potential of integrating TCD as a complementary or surrogate method for quantifying hemodynamic changes in studies that aim to calibrate or interpret BOLD‐fMRI data.

## FUNDING INFORMATION

This work was supported by Engineering and Physical Sciences Research Council UK through grant EP/S021507/1. GH was supported by Clarendon, and SS by the Rhodes Trust and the Canadian Institutes of Health Research (DSG‐193252).

## CONFLICT OF INTEREST STATEMENT

The authors declare that the research was conducted in the absence of any commercial or financial relationships that could be construed as a potential conflict of interest.

## Data Availability

The materials used to support the findings of this research are available from the corresponding author on reasonable request.
